# Outdoor public recreation spaces and social connectedness among adolescents

**DOI:** 10.1186/s12889-022-12558-6

**Published:** 2022-01-24

**Authors:** Elise Rivera, Jenny Veitch, Venurs H. Y. Loh, Jo Salmon, Ester Cerin, Suzanne Mavoa, Karen Villanueva, Anna Timperio

**Affiliations:** 1 Institute for Physical Activity and Nutrition (IPAN), School of Exercise and Nutrition Sciences, Deakin University, 221 Burwood Highway, Burwood VIC, Geelong, 3125 Australia; 2 Mary MacKillop Institute for Health Research, Australian Catholic University, Melbourne, Australia; 3 Melbourne School of Population and Global Health, The University of Melbourne, Melbourne, Australia; 4 The Centre for Urban Research, RMIT University, Melbourne, Australia

**Keywords:** Youth, Neighbourhood, Parks, Sports facilities, Beaches, Streets, Paths, Social interaction, Physical activity, Social connectedness

## Abstract

**Background:**

Outdoor public recreation spaces are important settings for leisure and physical activity. Adolescents’ use of these spaces may contribute to social connectedness via social interaction with peers and the community in these settings. However, research on this topic is limited. This exploratory study examined associations of frequency of visitation and physical activity in outdoor public recreation spaces with social connectedness among adolescents in Melbourne, Australia.

**Methods:**

Adolescents self-reported their frequency of visitation to parks, trails, beach/lake, and sports facilities; frequency of physical activity in a park, local street or path, and their street; and social connectedness. Separate analyses were conducted for visitation (*n* = 349, 15.4 ± 1.6 years, 58% female) and physical activity (*n* = 441, 15.4 ± 1.6 years, 59% female) using multilevel linear regression models.

**Results:**

No significant associations were observed for frequency of visitation to a park (B = 0.86, 95% CI = − 0.26, 1.99), trails (B = 0.41, 95% CI = − 0.61, 1.44), beach/lake (B = − 0.44, 95% CI = − 1.46, 0.57), or sports facilities (B = 0.64, 95% CI = − 0.43, 1.70), nor for frequency of physical activity in their street (B = − 0.07, 95% CI = − 0.46, 0.31), local street/path (B = − 0.05, 95% CI = − 0.43, 0.33) or in a park (B = 0.23, 95% CI = − 0.14, 0.60) with adolescents’ social connectedness.

**Conclusions:**

The findings did not support the hypothesis that visiting and being active in outdoor public recreation spaces are associated with adolescents’ social connectedness. Future research should consider the duration and context of outdoor public recreation space use (e.g., sports, socialising, relaxing alone) and whether different types and/or a combination of public spaces are more/less conducive to social connectedness.

## Introduction

Adolescence is a critical life stage characterised by profound changes in physiological, mental, and social development [1, 2]. In contrast to childhood, adolescents often have more social experiences [1] and spend more time engaging with people outside of the family sphere, such as friends [2, 3]; consequently, peer influence and the need for social connection increase [1, 4–6]. Social connectedness is defined as “the sense of belonging and subjective psychological bond that people feel in relation to individuals and groups of others” [7 , p. 1]. According to the self-psychology theory, social connectedness encompasses aspects of affiliation, companionship, and connectedness, and it is cultivated early in life and across the life course [8, 9]. During adolescence, social connectedness develops from the wide-ranging social interactions that adolescents have with people and their social environments [10–12]. Additionally, during adolescence, peer affiliations enable individuals to identify with others who have similar interests and appearances [9, 13], and this life stage involves complex social interactions as adolescents begin modelling behaviours on those of peers [14, 15].

Social connectedness is important for adolescent health as it can foster healthy development [16], reduce symptoms of anxiety and depression [17], and improve well-being [5, 18]. Social connectedness can also protect against the negative health impacts of social isolation [19] and loneliness [20]. Loneliness has been recognised as the lack of social connectedness and the perception of social isolation [18, 21], and it is a risk factor for high blood pressure [22], increased inflammation [23], depressive symptoms [24], reduced physical activity [25], and addiction [26]. It is therefore critical to promote social connectedness among adolescents.

According to Bronfenbrenner’s Ecological Systems Theory, social connectedness does not exist exclusively in a single setting (e.g., schools) but rather across multiple environments and contexts [27], such as peer, family, and neighbourhood or community [5, 28]. Within the neighbourhood context, outdoor public recreation spaces (e.g., parks, sports facilities) are places where people can gather and socialise with others, which may foster social connectedness. Evidence has indicated that adolescents value natural environments and outdoor public recreation spaces [29] for engaging in active and passive structured and unstructured leisure activities, such as socialising, running, free play, and sports [30, 31], which may support their social connectedness. Additionally, outdoor public recreation spaces, such as parks, paths, sports courts, streets, beaches, and skate parks located close to home have been shown to be especially important for adolescents [29, 30, 32, 33], as their ability to travel far from home may be limited. Further, these outdoor public recreation spaces are often freely accessible [34–36], and are located in many neighbourhoods in developed countries [35]. Outdoor public recreation spaces can provide meeting places for adolescents, and research has shown that adolescents who report having more opportunities for recreation and meeting people in their neighbourhood feel more connected with their neighbours [37]. However, little is known about the relationship between the use of outdoor public recreation spaces and adolescents’ social connectedness [38, 39]. Among adolescents, previous studies have largely focused on the influence of their experiences of neighbourhood destinations on their well-being [40] and physical activity [41], largely overlooking links with social connectedness [33, 42].

According to the conceptual model of the role of parks for public health by Bedimo-Rung and colleagues [34], parks and outdoor public recreation spaces can support social capital, which is interrelated with social connectedness [7], by providing a venue for people to socially interact and develop social ties. The model proposes that these social health benefits can be obtained in two ways: park visitation and active use of these settings (e.g., physical activity participation alone or with others upon arrival) [34, 39].

Firstly, qualitative evidence suggests that adolescents perceive parks, rivers, streets, and recreational facilities (e.g., sports fields/courts, skate parks) as appealing places to visit to “hang out” and socialise with friends [32, 33, 43]. Adolescents have reported being more likely to visit parks and open spaces that are used by their friends [44] and that are “popular” amongst peers [43, 45]. They also usually visit these settings in groups [46] and accompanied by others rather than visiting alone [47]. Additionally, adolescents have reported urban green spaces (e.g., parks) as being key places to meet new people and make friends [45, 48]. This suggests that adolescents’ visitation to outdoor public recreation spaces is largely driven by social factors, which may contribute to their social connectedness. However, additional research is warranted as one qualitative study has explored adolescents’ perceptions of how visiting parks can promote their social connectedness, with results showing that female adolescents viewed urban parks as supportive places for social interaction with peers and for forming social groups [43]. To our knowledge, no quantitative studies have examined associations between frequency of visiting outdoor public recreation spaces and adolescents’ perceived social connectedness.

Secondly, outdoor public recreation spaces may provide opportunities for adolescents to be active. A quantitative study found that, in comparison to other settings (e.g., home, school), adolescents spent significantly more time in moderate- to vigorous-intensity physical activity (MVPA) in green spaces (including parks) and in active transportation [49], which is typically performed on paths and streets. Adolescents have also reported “loose fit spaces” (e.g., paths, streets) as appealing for various (un)structured physical activities [32]. Qualitative and quantitative evidence has indicated that the most common physical activities that adolescents reported performing whilst visiting green spaces may involve social interaction and included playing sport, playing games, using equipment [48, 50], going for a walk/run, walking the dog, and riding a bike/scooter/skateboard [50].

Further, adolescents have previously reported being drawn to parks and recreational facilities where they could meet with other people and play sports due to the sense of camaraderie [51, 52], which may have important implications for their social connectedness. Group-based physical activity opportunities have also been reported by adolescents as being important avenues for making friends and cultivating a sense of belonging [53]. To our knowledge, no studies have examined whether the frequency of adolescents performing physical activity in their streets, local paths, and parks are associated with their social connectedness. Thus, this exploratory study sought to address the aforementioned research gaps by examining associations between frequencies of 1) visitation and 2) physical activity in outdoor public recreation spaces with social connectedness among adolescents. In alignment with the aforementioned conceptual model [34], we hypothesised that there would be positive associations between the frequencies of visiting and being active in outdoor public recreation spaces and adolescents’ social connectedness.

## Methods

### Study sample

Cross-sectional data from the NEighbourhood Activity in Youth (NEArbY) study were used. The NEArbY project is nested within a global study, the International Physical Activity and the Environment (IPEN) Adolescent project, which examined associations between the neighbourhood environment and physical activity among adolescents from 15 countries [54, 55]. Data were collected between August 2014–December 2015 from adolescents residing in Melbourne, Australia [56]. Ethical approvals were granted from the Deakin University Human Ethics Advisory Committee – Health (HEAG-H 152_2013). Approval for data collection in schools was granted by the Department of Education and Training (2013_002182) and the Catholic Education Office (Project #1950).

### School and participant recruitment

Adolescents were recruited from secondary schools selected from Statistical Area Level 1’s (SA1) across Melbourne [57], the smallest administrative unit used by the Australian Bureau of Statistics (ABS) to release census data [58]. Each SA1 in Melbourne was ranked by a walkability score [59] and by area-level income (derived from 2011 census data) [58]. SA1s were classified into four strata: high walkability/high income; high walkability/low income; low walkability/high income; low walkability/low income [57], and schools across these strata were approached.

Principals of each school were approached via a letter of invitation, followed by a phone call, to determine their interest in partaking in NEArbY. Those who expressed interest were sent a Plain Language Statement and written informed consent form. A total of 137 schools were approached, of which 18 consented and took part (response rate 13%): ten schools in high walkability/high income areas, three in high walkability/low income areas, five in low walkability/high income areas, and no schools in low walkability/low income areas.

Presentations were made to students in classes selected by the school, and recruitment packs (study information, informed consent forms, parent survey) were sent home with interested students. Written informed parental consent and student assent were required; 528 provided consent, 468 provided survey data, and of these, residential addresses were successfully geocoded for 465 [60]. Based on residential addresses, there was a relatively even spread of participants across the four SA1 strata: 23% in high walkability/high income areas, 28% in high walkability/low income areas, 25% in low walkability/high income areas, and 24% in low walkability/low income areas [57].

### Procedures and measures

Adolescents completed the survey on an iPad at school. The NEArbY survey was adapted from the IPEN adolescent survey [54] and was both constructed and administered using the online survey platform, Qualtrics.

#### Social connectedness

The Social Connectedness Scale was used to measure social connectedness as it has been shown to have an established internal reliability of 0.91 [8] and has been used in previous studies among adolescents [61, 62]. The Social Connectedness Scale includes items pertaining to belongingness (connectedness, affiliation, companionship), consistent with the self-psychology theory [8]. Adolescents were asked to indicate “how much do you agree or disagree with the following statements about your social connectedness?”: a) *I feel disconnected from the world around me*, b) *Even around people I know, I don’t feel that I really belong,* c) *I feel so distant from people,* d) *I have no sense of togetherness with my peers,* e) *I don’t feel related to anyone,* f) *I catch myself losing all sense of connectedness with society,* g) *Even among my friends, there is no sense of brother/sisterhood,* and h) *I don’t feel that I participate with anyone or any group* [8]*.* A four-point Likert scale (1 = strongly disagree to 4 = strongly agree) was used to indicate the level of agreement with the items. Responses were reverse-coded and summed to determine an overall social connectedness score (possible range: 8–32) with a higher score indicating a higher degree of social connectedness [8]. In the current sample, the internal consistency of the scale was high (Cronbach’s alpha: 0.93), which is similar to previous research [8].

#### Frequency of visitation to local outdoor public recreation spaces

Participants were asked to report (open response field) how frequently they visited (number of times/usual week) their nearest: a) small public park; b) large public park; c) bike/hiking/walking trails, paths; d) beach, lake, river or creek; e) basketball court (e.g., full court, ½ court); and f) other public playing fields/courts (e.g., soccer field, skate park). Survey items were adapted from the Neighbourhood Environment and Walkability Scale for Youth [63], and similar items have been shown to have acceptable test-retest reliability (ICC range 0.39–0.66) [64, 65]. A composite score for visitation to a public park was created by summing responses for a small and a large public park. Similarly, a composite score was created for public sports features/facilities by adding responses for a basketball court and for other playing fields/courts (e.g., soccer field, skate park). Due to the high number of participants who reported not visiting the different outdoor public recreation places in a usual week, responses were dichotomised as (coding in parentheses): visit less than once in a usual week (0) and visit at least once in a usual week (1). The open response field in the survey did not prevent written responses, which were provided by many participants. Where applicable, relevant written responses were also coded as 0 or 1. Examples of responses coded as ‘visit less than once in a usual week’ were ‘not often’, ‘sometimes’, ‘not much’, ‘never’ and as ‘visit at least once in a usual week’ were ‘yes’, ‘often’, ‘usually’ and ‘always’.

#### Frequency of physical activity in local outdoor public recreation spaces

Participants reported how frequently they engaged in physical activity in: a) their street; b) on a local street or footpath/bike path; and c) in a nearby park/reserve. These items were adapted from previous research [66], and similar items have been shown to have acceptable test-retest reliability (ICC range 0.31–0.82) [64]. The original categorical responses in the survey were: never; once a month or less; once every other week; once/week; 2–3 times/week; and 4 or more times/week. These were recoded as continuous to reflect the frequencies of the response options (coding in parentheses): never (0); once a month or less (0.25); twice per month (0.5); once/week (1); 2–3 times/week (2.5); and 4 or more times/week (4).

#### Demographics

Adolescents reported their birth date and sex (male, female). Missing information regarding age was obtained from a parent survey (*n* = 7).

### Data analysis

Complete case analyses were performed separately for the two predictors, frequency of visitation and physical activity in outdoor public recreation spaces. Thus, two samples were analysed according to the outcome, social connectedness. For analyses using frequency of visitation, we excluded 119 participants with missing data for the following variables: social connectedness (*n* = 17); frequency of visitation to a public park (*n* = 59), trails/paths (*n* = 78), beach/lake (*n* = 92) and sports features (*n* = 96); age (*n* = 9); sex (*n* = 11); and SA1 of participants (*n* = 3). This reduced the sample from 468 to 349 participants. For analyses using frequency of physical activity, we excluded 27 participants with missing data for the following variables: social connectedness (*n* = 17); frequency of physical activity in participants’ street (*n* = 6), local street or path (*n* = 7), and park/reserve (*n* = 7); age (*n* = 9); sex (*n* = 11); and SA1 of participants (*n* = 3). This reduced the sample from 468 to 441 participants. Complete case analyses and descriptive analyses on the predictor and outcome variables were performed using Stata/SE 16.0 (Stata Corp., College Station, TX, USA).

We used different types of regression models to test for curvilinearity and selected the model with best fit to the data for analyses. Multilevel linear regression models were used to examine associations of frequency of visitation and frequency of physical activity in each of the locations with social connectedness. All models specified school and SA1 as random effects to account for cross-clustering and adjusted for age and sex. Significance was set at *p* < 0.05.

Due to the high proportion of missing responses for frequency of visitation, sensitivity analyses were performed to determine whether there were differences for those with missing data versus without missing data for the predictor variables according to age, sex, and social connectedness. These analyses (not presented here) revealed that social connectedness was negatively associated with having missing data [OR = 0.95, (95%CI = 0.95, 1.28]), *p* = 0.024].

## Results

Results from the descriptive analyses and complete case analyses on the predictor and outcome variables are presented below.

### Frequency of visitation to local outdoor public recreation spaces

As shown in Table 1, on average, participants were 15 years of age (range 12 to 18 years); 58% were female; and at least 50% visited the nearest public park, trail/path, beach/lake, or sports features at least once in a usual week. The average social connectedness score of participants was 28. No statistically significant associations between frequency of visitation to any of the examined locations and adolescents’ social connectedness scores were observed (Table 2).

**Table 1:** Frequency of visitation to and physical activity in outdoor public recreation spaces

Visitation (*n* = 349)	
Age (years), mean (SD)	15.4 (1.6)
Sex	
Female, n(%)	201 (57.6)
Frequency of visitation to a public park n(%)	
Visit at least once/week	256 (73.4)
Visit < once/week	93 (26.7)
Frequency of visitation to a trail/path, n(%)	
Visit at least once/week	198 (56.7)
Visit < once/week	151 (43.3)
Frequency of visitation to beach/lake, n(%)	
Visit at least once/week	216 (61.9)
Visit < once/week	133 (38.1)
Frequency of visitation to sports features (basketball, other courts, fields, skate park), n(%)	
Visit at least once/week	233 (66.8)
Visit < once/week	116 (33.2)
Social Connectedness Score, mean (SD)	28.2 (4.8)
**Physical activity (n = 441)**	
Age (years), mean (SD)	15.4 (1.6)
Sex	
Female, n(%)	259 (58.7)
Frequency of physical activity in own street, n(%)	
Never	139 (31.5)
Once a month or less	83 (18.8)
Twice per month	55 (12.5)
Once a week	78 (17.7)
2–3 times/week	54 (12.2)
> 4 times/week	32 (7.3)
Frequency of physical activity in a local street or footpath/bike path, n(%)	
Never	146 (33.1)
Once a month or less	71 (16.1)
Twice per month	55 (12.5)
Once a week	67 (15.2)
2–3 times/week	62 (14.1)
> 4 times/week	40 (9.1)
Frequency of physical activity in a nearby park/reserve, n(%)	
Never	83 (18.8)
Once a month or less	76 (17.2)
Twice per month	71 (16.1)
Once a week	92 (20.9)
2–3 times/week	81 (18.4)
> 4 times/week	38 (8.6)
Social Connectedness Score, mean (SD)	27.9 (4.9)

Note: possible range for Social Connectedness score: 8–32 (32 is maximum score)

**Table 2:** Associations between visitation to and physical activity in outdoor public recreation spaces with social connectedness score

	Social Connectedness Score	
**Locations for visitation (*n*** **= 349)**	**B (95%CI)**	***p*-value**
Public park	0.86 (−0.26, 1.99)	0.132
Trail/path	0.41 (−0.61, 1.44)	0.426
Beach/lake	−0.44 (−1.46, 0.57)	0.392
Sports features (basketball, other courts/fields, skate park)	0.64 (−0.43, 1.70)	
**Locations for physical activity (*n*** **= 441)**	**B (95%CI)**	***p*-value**
Participant’s street	−0.07 (−0.46, 0.31)	0.705
Local street or footpath/bike path	−0.05 (− 0.43, 0.33)	0.788
Nearby park/reserve	0.23 (−0.14, 0.60)	0.225

### Frequency of physical activity in local outdoor public recreation spaces

On average, participants were 15 years of age (range 12 to 18 years); 59% were female; the average social connectedness score was 28; and less than 50% of the sample were active on their street, on a local street/path, or in a nearby park at least once in a usual week (Table 1). No statistically significant associations were observed between frequency of physical activity in their street, on a street or bike/foot path, or at a nearby park/reserve and adolescents’ social connectedness score (Table 2).

## Discussion

This exploratory study examined associations between the frequencies of visiting and being active in certain outdoor public recreation spaces with social connectedness among adolescents. The findings indicated no associations for frequency of visitation to the nearest public park, trails/path, beach/lake/river/creek, or sports features (e.g., basketball court, soccer field). Additionally, no associations were observed for frequency of physical activity in participants’ street, on a local street or bike/foot path, or at a nearby park/reserve with social connectedness.

Contrary to our hypothesis, we found limited evidence for associations between visiting outdoor public recreation spaces in a usual week and social connectedness among adolescents. Participants reported frequency of visitation to the nearest outdoor public recreation spaces to their homes. While at least half of the sample reported visiting the locations at least once in a usual week, it could be that adolescents also visited outdoor public recreation spaces located further away from home that attracted their peers to “hang out” and socialise. Previous qualitative evidence among adolescents has indicated that parks in a convenient location near school, friends’ houses, shops, and public transport would encourage visitation [47], and adolescents have reported that they would be prepared to visit high-quality parks located further away from home [47, 67]. Future studies should therefore consider examining adolescents’ visitation to the closest and the most frequently visited outdoor public recreation spaces in their neighbourhood. It is also unknown what activities adolescents were engaging in whilst in the settings, and it is possible that these activities may not have involved social interaction. A further reason for the limited evidence of associations between visiting outdoor public recreation spaces and social connectedness may be that we did not assess other public settings that are also important for adolescents’ social connectedness, such as plazas, civic squares and shopping strips [31, 32, 68]. In addition, we did not measure duration of visits to outdoor public recreation spaces, so it could be that adolescents in our sample were not spending sufficient time at the locations for there to be significant associations with social connectedness.

Previous qualitative research has found that while the presence of other people attracts adolescents to visit urban parks, minimal interaction between different user groups was often reported, and most adolescents kept to their social groups [69]. This suggests that co-presence with other user groups may not translate into meaningful contact, which may have been the case in the present study. Thus, although outdoor public recreation spaces provide opportunities for incidental interactions [70, 71], it could be that adolescents who visited their nearest public parks, beach/lake, trails, and sports features at least once in a usual week were not experiencing the types of social interactions necessary for fostering social connectedness. Moreover, Gibson’s Affordance Theory postulates that environments afford various behaviours and actions [72], and the perceived opportunities for use are relational to the needs and interests of users in the environment [73, 74]. Our findings may also be explained by the likely variation in adolescents’ needs and interests. Given that outdoor public recreation spaces support many different activities (not limited to social interaction and physical activity), future research may benefit from examining how affordances and context of use of these settings influence adolescents’ social connectedness.

Our findings also showed that there was limited evidence of associations between the frequency of performing physical activity in the examined outdoor public recreation spaces and social connectedness, and there are several possible explanations for this. Firstly, at least half of the sample did not visit a nearby park, their street, or a local street or path at least once per week, so it may be that they were not active frequently enough at these places for there to be significant associations with social connectedness. Additionally, regarding streets and paths, it could be that these areas may be used more often for active transportation [75, 76] – walking, running, and/or cycling for travel [76] – than for active recreation. It is possible that active travel on streets/paths (even if accompanied) may be less conducive to the social interactions needed to foster adolescents’ social connectedness. Further, evidence has indicated that adolescents engage in a range of physical activities in parks, such as organised sports, playing games and/or on play equipment, walking, and running [48, 50]. Given that adolescents value opportunities for being active while socialising with peers [77, 78], it could be that the specific types of physical activities undertaken, as well as whether they involve accompaniment and interaction with others (e.g., organised sports versus walking alone), matter for supporting social connectedness. Future research should consider exploring associations between types of physical activity undertaken in outdoor public recreation spaces with social connectedness among adolescents.

The Social Connectedness Scale was used in this exploratory study, and the scores of adolescents in our sample were comparable to scores in other studies, which have used modified versions of the same scale with adolescents [61] and young adults [8, 79]. However, there is currently no universal instrument for assessing social connectedness and other measures are available [7, 13, 18, 80, 81]. It has also been recognised that adolescents develop social relationships across multiple environments [27, 82], and social connectedness can be both experienced in and cultivated across different social domains/contexts (e.g., school, family, community/neighbourhood, peer) [5, 18, 28]. For instance, it is possible that adolescents may feel socially connected in the peer context, but not in the neighbourhood setting. Additionally, social connectedness has an autonomy aspect, where individuals feel they are valued within groups and relationships, and a relational aspect where there is a connection to others [80]. Therefore, even if adolescents interact socially with people, if they do not feel valued and validated by these bonds, then this may impact how socially connected they feel [80]. This poses challenges when trying to accurately assess social connectedness. The limited evidence of associations observed may have been due to these challenges. In addition, the scale employed in this study was a general measure of social connectedness rather than of social connectedness specifically in the community. However, to our knowledge, there is no context-specific measure for social connectedness, so this is an area for further investigation. Moreover, when seeking to understand people-place relationships, place-related constructs (e.g., sense of community, sense of place, place attachment) have commonly been used [83–85]. Future research may also benefit from examining place constructs in relation to adolescents’ use of outdoor public recreation spaces.

To our knowledge, this is the first study to examine associations of frequencies of visitation to and physical activity in outdoor public recreation spaces with adolescents’ social connectedness. Additional strengths include the examination of multiple outdoor public recreation spaces and the roughly equal split of males and females in the analytical sample. However, as a cross-sectional study that relied on self-report, causal inferences between variables cannot be drawn, and the data may be subject to recall or social desirability bias [86, 87]. Future studies may consider using ecological momentary assessment to sample and examine participants’ social experiences and behaviours in real time [88] or including measures of social interaction within outdoor public recreation spaces, whether physical activity in these settings was performed with others, and mode of travel to these settings to more explicitly address the research question. Further, responses were dichotomised as visiting less than once versus at least once in a usual week. It is also possible that participants considered both indoor and outdoor sports features when completing the items regarding the nearest basketball court and other fields/courts. Future research may also benefit from using objective measures, such as accelerometers or direct observation [89], for assessing visitation and physical activity in outdoor public recreation spaces in addition to assessing the duration and context of use. Additionally, the present study performed secondary analyses of data collected from 2014 to 2015, so the findings and conclusions may not be applicable to the context of the COVID-19 pandemic.

Furthermore, the limited evidence of associations between the predictor and outcome variables may have been due to statistical reasons. There was a high proportion of missing responses for the predictor variables (particularly visitation to different outdoor public recreation spaces). While complete case analyses are commonly used to address missing data in epidemiological research [90], this approach can bias findings and lead to losses in statistical power and precision [90, 91]. The analyses of patterns of missingness performed showed that data were at least ‘Missing at Random’ (MAR) [90]. Specifically, adolescents with a higher social connectedness score were less likely to have missing data compared to those with lower social connectedness. Consequently, our findings may have been biased due to the lack of power to detect associations resulting from the reduced analytical sample [90, 91]. Additionally, when data are not ‘Missing Completely At Random’ (MCAR), complete case analyses can bias regression coefficients. This bias increases with the difference between means of the observed and missing cases and with the proportion of missing cases [92].

## Conclusion

The findings of this exploratory study provide limited evidence of associations between visiting and being active in outdoor public recreation spaces and social connectedness among this particular sample of adolescents. Future research should consider the time that adolescents spend in outdoor public recreation spaces, the context of use, and adolescents’ social connectedness specifically in the neighbourhood or community domain. Future studies may also benefit from exploring the influence of different types of activities undertaken in outdoor public recreation spaces and whether accompaniment in these settings is important for supporting social connectedness among adolescents.

## Data Availability

The dataset analysed during the current study are not publicly available due to ethical restrictions related to the consent given by participants at the time of study commencement. An ethically compliant dataset is however available from the corresponding author on reasonable request and with approval of the Deakin University Human Research Ethics Committee.

## Abbreviations

ABS: Australian Bureau of Statistics
MVPA: Moderate- to Vigorous- Physical Activity
NEArbY: NEighbourhood Activity in Youth project
IPEN: International Physical Activity and the Environment
SA1: Statistical Area Level
